# Legal Complexities of Adolescent Relationships: A Study of Protection of Child Sexual Offense Cases in India

**DOI:** 10.7759/cureus.60475

**Published:** 2024-05-17

**Authors:** Srushti Shukla, Preeti Tiwari, Arijit Datta, Swapnil Agaarwal, Dhara Goswami, Darshan D Galoria

**Affiliations:** 1 Forensic Medicine, Pramukhswami Medical College & Sri Krishna Hospital, Bhaikaka University, Karamsad, IND; 2 Community and Family Medicine, Pramukhswami Medical College & Sri Krishna Hospital, Bhaikaka University, Karamsad, IND; 3 Forensic Medicine, Gujarat Adani Institute of Medical Sciences, Bhuj, IND

**Keywords:** adolescent, indian legal system, consensual sexual act, sexual assault, protection of children against sexual offences (pocso) act

## Abstract

Introduction

The Protection of Children from Sexual Offences (POCSO) Act in India prohibits sexual engagement among individuals under 18 years old. However, societal variables also affect many teenagers’ consensual sexual activities. Little research has been conducted on the legal ramifications for consenting to sexual action under the POCSO Act. This study examined consensual sexual assault cases under the POCSO Act and their possible outcome.

Methodology

Five years of medicolegal records from a tertiary hospital were analyzed for sexual assault victims aged <18 years. Descriptive statistics were used to analyze victim demographics, literacy, sexual acts, reporting patterns, and accused-victim relationships.

Result

A total of 410 instances of sexual assault were recorded, and 29% involved victims between the ages of 16 and 18. Most victims (73.9%) in this age range were literate, and 85.7% provided consent for sexual relations. Parents or guardians reported all cases, and most of the accused were victims' friends.

Discussion

The POCSO Act offers legal safeguards for sexual abuse and exploitation. However, their use in adolescent sexual consent has raised concerns. The Law Commission of India's refusal to reassess the POCSO Act's age barrier for sexual consent emphasizes the need for a child-centric approach to negotiating complex teenage relationships.

Conclusion

Balancing legal obligations and developmental needs is essential to addressing consensual sexual acts under the POCSO Act. While sensitively implementing the law, stakeholders must focus on their children's best interests and healthy development. Child-friendly environments and support systems empower victims and reduce trauma in teenage relationships.

## Introduction

Romantic relationships and sexual instincts are crucial developmental phases, particularly during adolescence. In India, the Protection of Children from Sexual Offences Act, 2012 (POCSO Act) prohibits sexual engagement in romantic relationships within the age of 16-18 years [1]. However, owing to sociocultural influences, many adolescents run away with their partners to secure relationships. There is limited literature on the legal implications of cases registered in India, and there is no literature available that exclusively discusses the legal implications of consensual sexual acts from a legal perspective [2,3]. The presence of this study gap underscores the need for further research on the legal difficulties encountered by adolescents participating in consensual sexual activities in the context of the POCSO Act [4]. Understanding the legal framework of teenage relationships can provide valuable insights for the development of policies and interventions to safeguard vulnerable children. Although it has been about 10 years since the ratification of this act, there is a scarcity of studies in this field that primarily concentrate on the implementation and functioning of the act [5]. In this study, we analyzed sexual assault victim examination certificates between the age of 16 and 18 years in the last five years from a tertiary healthcare facility situated in the district of Gujarat. Interestingly, while the act is designed to protect children, it may inadvertently impact adolescents' sexual rights by potentially criminalizing consensual sexual activities among them because of the strict age of consent laws. This could lead adolescents to avoid seeking help or information about sexual health for fear of legal outcomes. We also discuss how the POCSO Act influences adolescents' sexual rights, and the need to evaluate its implications.

## Materials and methods

This study was conducted after obtaining approval from the institutional ethics committee (IEC/BU/2022/Ex. 61/258/2022). The researchers adhered to rigorous protocols to safeguard the well-being and privacy of participants during the study.

Sample calculation

The investigation covered all incidents documented between January 1, 2019, and December 31, 2023.

Study parameters

The dataset included information on the victim's age, gender, literacy level, type of sexual act committed (consensual or non-consensual), the accused's familiarity with the victim, and the individual who reported an alleged sexual assault. The age and sex of the victims were recorded while they completed their medicolegal certificate. According to the Government of India guidelines, if a person can read and write with an understanding of any language, they will be considered literate [6]. If the victim read the consent form and gave consent for the medicolegal examination by signing, then she would be considered literate; if she used a thumbprint to give consent, then she would be considered illiterate; information regarding the accused's familiarity with the victim and consent for the alleged sexual act was recorded during the process of taking history; and finally, information regarding the complainant was gathered from the police. Victims who presented with a history of sexual assault at the Clinical Forensic Medicine unit of a tertiary healthcare center and who provided consent for medicolegal examination were included in the study. The study did not include victims who refused to undergo medicolegal examination and were referred from a primary health center for medical treatment purposes. No additional investigations or interventions were introduced to the victims. No data (identifiers) revealing victims’ identifications were noted.

Statistical analysis

Excel sheets were used as the initial input and data arrangement. This adaptable tool enabled the organization of unprocessed data and generation of appropriate datasets for the study. Statistical Package for the Social Sciences software version 22.0 (IBM Corp., Armonk, NY, IBM Corp) was used to conduct descriptive statistical analyses. The collected data were stored with appropriate safety measures and were only accessible to the investigators. Strict protocols were followed to ensure the confidentiality and security of the data. This information is encrypted and regularly backed to prevent unauthorized access.

Study design

This is a record-based study. Data were collected from the medicolegal records and no identifiers were recorded.

## Results

**Figure 1:**
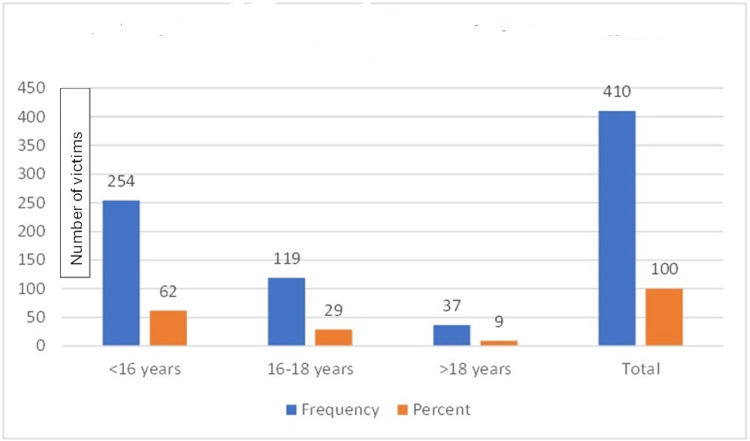
Age distribution of reported cases

In the duration of five years, 410 cases were examined. Among these cases, the age of the victims was between 16 and 18 years in 119 instances, while 254 cases had victims aged <16 years. Figure 1 depicts the age distribution among the total number of reported cases. The findings also revealed that in all 119 cases of alleged sexual assault where the victim's age was between 16 and 18 years, parents or guardians reported the cases to the police and not the victim or her friends.

**Figure 2:**
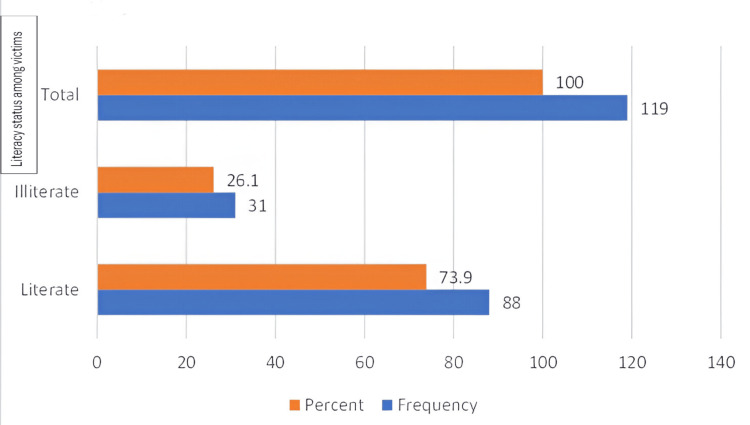
Literacy status among victims of age group 16-18

Among the 119 instances where the victims were between 16 and 18 years of age (n=88), 73.9% were literate, suggesting that most alleged victims were educated and knowledgeable about their rights. Figure 2 depicts the literacy status among a total number of reported cases.

**Figure 3:**
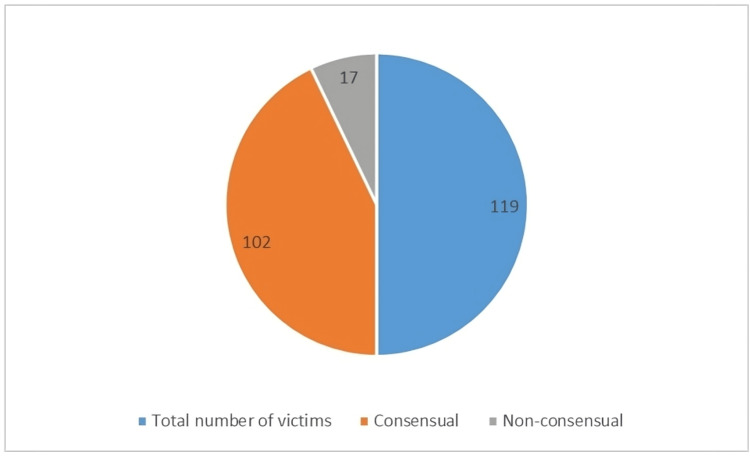
Consensual/Non-consensual sexual intercourse among victims of age group 16-18

The findings indicated that of the 119 individuals aged 16-18 who were surveyed (n=102), 85.7% agreed to engage in sexual activity with their partners. In contrast, only (n=17) 14.3% reported experiencing non-consensual sexual activity. These findings provide insight into the dynamics of alleged sexual assault within the study population, emphasizing the prevalence of consensual encounters. Figure 3 depict about consensual and non-consensual sexual intercourse among the age group of 16-18.

The findings also revealed that in all 119 cases of alleged sexual assault where the victim's age was between 16 and 18 years, parents or guardians reported the cases to the police and not the victim or her friends. Table 1 depicts the person who reported the sexual assault to police.

**Table 1:** Reporting of sexual assault by victim or guardian/relatives

Reporting of Sexual Assault in the Age Group of 16-18 Years	Frequency	Percent
Guardian/relatives	119	100.0
Victim herself	0	0
Total	119	100.0

Table 2 illustrates the relationship between the accused and victim among the 119 cases of alleged sexual assault. It showed that (n=118) 99.2% of the cases involved an accused in a friendly relationship with the victim, and the remaining 0.8% (n=1) involved a victim who did not know the accused before the alleged incident.

**Table 2:** Relationship between alleged accused and victim

Familiarity between the Alleged Accused and Victim	Frequency	Percent
Friends	118	99.2
Strangers	1	0.8
Total	119	100.0

## Discussion

The POCSO Act of 2012 is comprehensive legislation in India that aims to safeguard children from all types of sexual abuse. This act was enacted under Article 15(3) of the Constitution of India, and it recognizes a child's best interest, defined as any person under the age of 18. The Preamble emphasizes the importance of securing the child's best interests and ensuring their healthy, physical, emotional, intellectual, and social development. The POCSO Act of 2012 was enforced on November 14, 2012, owing to the efforts of various non-governmental organizations, activists, and the Ministry of Women and Child Development. It covers seven types of sexual offenses: penetrative sexual assault, aggravated penetrative sexual assault, sexual assault, aggravated sexual assault, sexual harassment, use of a child for pornographic purposes, storage of pornographic materials involving a child, attempt/abetment, media violation of the Indian Penal Code (IPC) 228 (A), false complaints, failure to report, failure to record cases, and offenses under other acts. The POCSO Act provides strict punishments for the commission of offenses against children, ranging from a minimum of 20 years of imprisonment to the death penalty in the case of aggravated penetrative sexual assault. Before the introduction of the POCSO Act, child sexual abuse accounted for offenses under Sections 375, 354, and 377 of IPC 1860. However, they do not protect male children from sexual abuse or modesty. The POCSO Act provides legal protection against sexual abuse and exploitation, establishes special courts for speedy trials and incorporates child-friendly procedures. It emphasizes preventive measures, imposes severe penalties on offenders, and mandates support services for victims. The act empowers children and encourages the reporting of abuse, ensuring that no form of abuse goes unrecognized or unpunished [1,7]. Undoubtedly, the POCSO Act of 2012 is a significant and ultimate law in India that effectively protects the rights and well-being of children through a comprehensive and well-defined framework [8]. According to the National Crime Records Bureau (NCRB) 2022, total number of child victims were 64469 and crime rate per lakh population was 14.3 [9]. Previous research conducted by the Center for the Child and Law at the National Law School University in India and other agencies has shown that the age group of 16-18 years old had the most recorded instances. Studies conducted by these organizations indicate a significant number of cases within this age bracket [10]. Michael Foucault identifies two types of regulatory power in society: juridico-discursive power, which is formal and unified, and disciplinary power, which is informal and fragmented. Juridico-discursive power is applied aggressively and authoritatively, whereas disciplinary power is used to maintain social norms and order in institutions such as schools, hospitals, and prisons by regulating individuals' behavior. Disciplinary power focuses on surveying behavior within these institutions, while juridico-discursive power involves enforcing rules through legal and punitive measures. These two forms of power collaborate to control and shape the behavior of those within these institutional settings, with disciplinary power operating within the context of law and state authority to uphold social norms and order. By following these principles, Sutherland examined the age of consent provision in US criminal laws in the 1990s to prosecute cases of consensual sex among underage girls. The focus of prosecution was often on preventing teenage pregnancies and minimizing the financial burden on the government [11]. However, it seemed that the law was not primarily concerned with protecting young girls from sexual assaults. Instead, cases were selectively brought against boyfriends when their teenage partners applied for welfare support. If a boyfriend or father was willing to accept responsibility, cases were often dismissed or dealt with leniently. However, if the sex of underage girls was consensual and did not result in pregnancy, or if it occurred within the middle or upper classes, it was less likely to lead to welfare payments [12]. The Law Commission of India aims to reform laws to maximize justice and promote good governance under the rule of law. Its mission includes reviewing obsolete laws; examining socioeconomic legislation and the judicial administration system; examining existing laws; considering state policy directives; suggesting improvements and reforms; and proposing legislation to achieve the objectives set out in the Constitution. The commission also examines existing laws for gender equality and suggests amendments, revising central acts to simplify them and removing anomalies. The commission's vision is to ensure responsiveness to the demands of the times and to protect the marginalized. However, in the case of the POCSO Act, the law commission refused to tinker with existing rules to provide consent for sexual intercourse [13]. According to the present study, a majority of the 119 individuals reported as victims (73.9 %) were literate, indicating that they were aware of their situation. This finding is higher than the results reported by Rani et al. [14] and Verma et al. [15], where 36.6% and 74.14% of victims were educated, respectively. Our study also showed that 85.7% of females (n=102) consented to engaging in sexual activities with their partners, whereas only 14.3% (n=17) were involved in non-consensual sexual encounters. This is similar to the findings of Bhoi et al., where 41.68% of cases in their study were consensual [16]. Our investigation found that, in every instance where consensual sexual activity occurred between individuals aged 16 and 18 years, the incidents were reported by their parents, which has not been addressed in any other study. Additionally, our research revealed that in 119 cases of reported sexual assault on individuals aged 16-18 years, the alleged accused was in a friendly relationship with the alleged victim. This contrasts with the study by Lal et al. where the accused was familiar to the victim in 80.9% of cases [17]. Notably, only one of the cases involved a stranger as the accused. Historically, marriage has been viewed as a social duty, whereas love has been celebrated as a personal ideal. Marriage is the arrangement of political alliances and economic exchanges based on duty. Love is believed to transcend one's life; however, romantic love is only considered acceptable if it leads to marriage to a partner from an appropriate class, caste, and religion. However, today, India is becoming more lenient in its attitude toward love, particularly in urban areas. Upper-class Indian youth have creatively reconciled this dichotomy by embracing individualism and romantic love in their cultural heritage. Authors recommend that law commission must consider to lower the age of consensual sexual intercourse.

Limitations

More elaborate, countrywide research is beneficial, as this study's findings are limited to a particular district in Gujarat.

## Conclusions

In India, teenage romantic relationships are influenced by a multitude of sociocultural factors. From a legal perspective, the issue of consent continues to be contentious as outlined in the 2012 POCSO Act. This involves balancing age-appropriate developmental requirements and legal responsibilities of children in India. Regrettably, instances of child sexual assault were overshadowed by this argument. At this point, all parties must seriously consider these concerns and work toward creating a child-friendly environment. This measure will effectively facilitate the amplification of victims' voices and mitigate the unnecessary trauma experienced by adolescent couples engaged in romantic relationships. Ultimately, ensuring that the POCSO Act is implemented with sensitivity and an understanding of the complexities of adolescent relationships is crucial. Young individuals at the bottleneck of adulthood must be supported and guided to navigate such complex situations, and should not be branded as victims or accused.
